# The Effects of Dietary Iron and Capsaicin on Hemoglobin, Blood Glucose, Insulin Tolerance, Cholesterol, and Triglycerides, in Healthy and Diabetic Wistar Rats

**DOI:** 10.1371/journal.pone.0152625

**Published:** 2016-04-11

**Authors:** Adriana Márquez-Ibarra, Miguel Huerta, Salvador Villalpando-Hernández, Mónica Ríos-Silva, María I. Díaz-Reval, Humberto Cruzblanca, Evelyn Mancilla, Xóchitl Trujillo

**Affiliations:** 1 Unidad de Investigación Dr. Enrico Stefani, Centro Universitario de Investigaciones Biomédicas, Universidad de Colima, Col. Villas San Sebastián, Colima, Colima, México; 2 Centro de Investigación en Nutrición y Salud, Instituto Nacional de Salud Pública, Universidad No. 655 Colonia Santa María Ahuacatitlán, Cerrada Los Pinos y Caminera C.P., Cuernavaca, Morelos, México; Monash University, AUSTRALIA

## Abstract

**Objective:**

Our aim was to assess the effects of dietary iron, and the compound capsaicin, on hemoglobin as well as metabolic indicators including blood glucose, cholesterol, triglycerides, insulin, and glucose tolerance.

**Materials and Methods:**

Our animal model was the Wistar rat, fed a chow diet, with or without experimentally induced diabetes. Diabetic males were fed control, low, or high-iron diets, the latter, with or without capsaicin. Healthy rats were fed identical diets, but without the capsaicin supplement. We then measured the parameters listed above, using the Student t-test and ANOVA, to compare groups.

**Results:**

Healthy rats fed a low-iron diet exhibited significantly reduced total cholesterol and triglyceride levels, compared with rats fed a control diet. Significantly reduced blood lipid was also provoked by low dietary iron in diabetic rats, compared with those fed a control diet. Insulin, and glucose tolerance was only improved in healthy rats fed the low-iron diet. Significant increases in total cholesterol were found in diabetic rats fed a high-iron diet, compared with healthy rats fed the same diet, although no statistical differences were found for triglycerides. Hemoglobin levels, which were not statistically different in diabetic versus healthy rats fed the high-iron diet, fell when capsaicin was added. Capsaicin also provoked a fall in the level of cholesterol and triglycerides in diabetic animals, versus diabetics fed with the high iron diet alone. In conclusion, low levels of dietary iron reduced levels of serum triglycerides, hemoglobin, and cholesterol, and significantly improved insulin, and glucose tolerance in healthy rats. In contrast, a high-iron diet increased cholesterol significantly, with no significant changes to triglyceride concentrations. The addition of capsaicin to the high-iron diet (for diabetic rats) further reduced levels of hemoglobin, cholesterol, and triglycerides. These results suggest that capsaicin, may be suitable for the treatment of elevated hemoglobin, in patients.

## Introduction

The possibility that iron plays a role in the development of diabetes has been suggested by several authors [1]. Multiple recent studies have shown that iron accumulation increases the risk of developing type-2 diabetes mellitus, with its depletion shown to be protective [1–3]. Mechanistically, the major iron-regulating hormone, hepcidin, is known to regulate intestinal iron absorption, and modulates erythropoiesis [4]. Also of relevance is the renal dysfunction that accompanies diabetes, which can also modify hemoglobin regulation [5,6].

The increased consumption of dietary iron leads to elevated iron storage in the body, bound by the protein, ferritin. Increased iron storage correlates with the development of diabetes [7,8], with dietary iron overload shown to interfere with both plasma lipid transport (which increases triglyceride and cholesterol levels), and blood lipids (in Sprague Dawley rats [9,10]). In mice, increased dietary iron induces insulin resistance, and elevated levels of hepcidin [11]; iron depletion, achieved using phlebotomy, iron chelators, or a low-iron diet, increases insulin sensitivity, and secretion [12]. In addition, reduced iron intake, or the depletion of iron stores, improves the lipid profile; an effect that can be reversed by high levels of dietary iron, which also adversely influences total lipid level [13–15]. Interestingly, the vanilloid receptor 1 (also termed the capsaicin receptor, or the transient receptor potential cation channel subfamily V member 1 (TRPV1)), participates in the regulation of pancreatic beta cell function [16], with capsaicin itself acting to enhance intestinal iron uptake in experiments in vitro[17], which, of itself, promotes a potent antioxidant effect [18].

This study aimed to determine whether low- or high-iron diets, the latter with or without capsaicin, could affect insulin tolerance, levels of glucose, cholesterol, triglycerides, and hemoglobin. For this investigation, we used healthy and diabetic Wistar rats.

## Materials and Methods

### Animals

**Fig 1:**
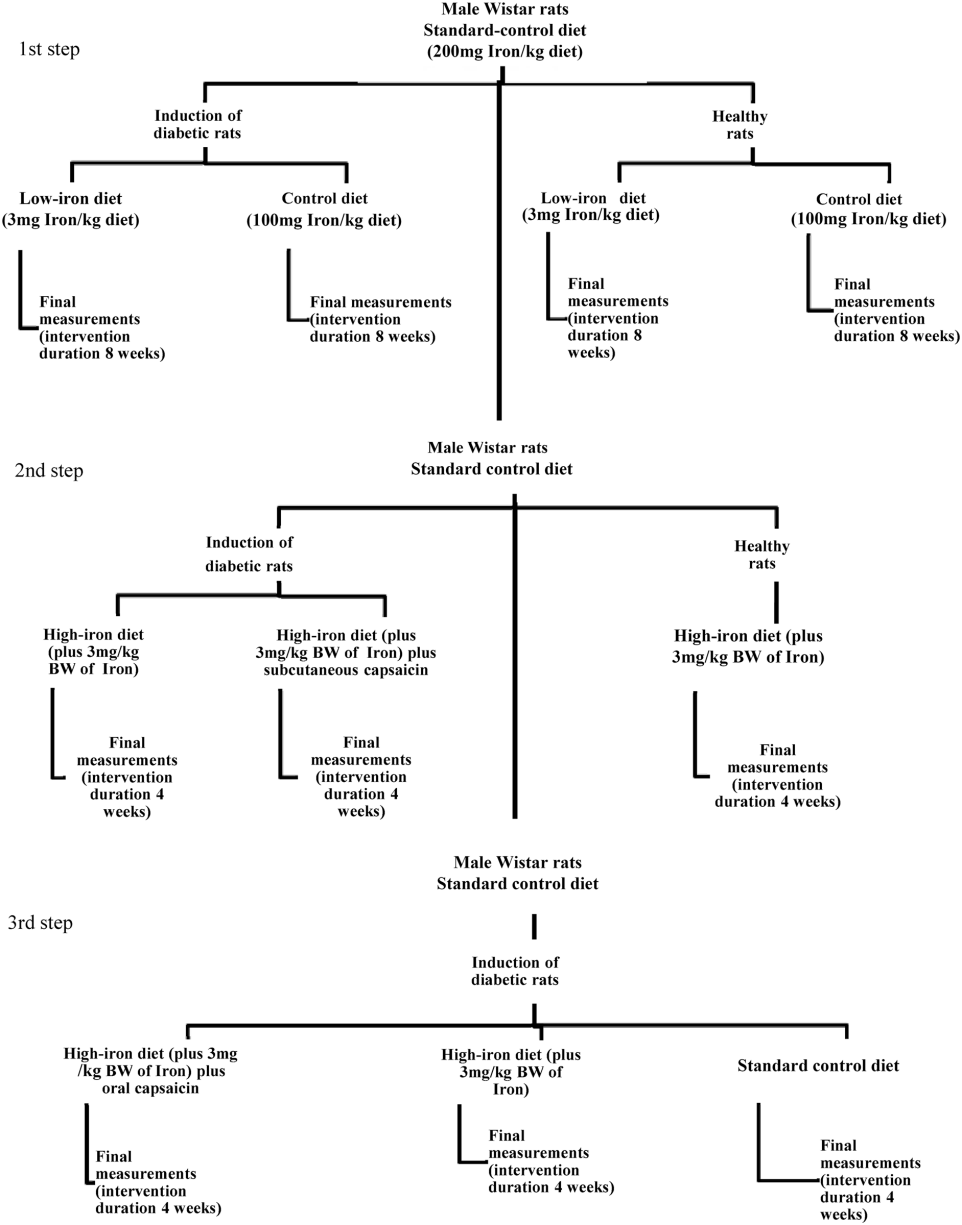
Study design.

Male Wistar rats (Harlan Laboratories, Inc. Stoughton Rd, USA), weighing 250 to 350 g each, and aged approximately two months, were split into several randomized groups. All animals were maintained at the Laboratory Animals of the Centro Universitario de Investigaciones Biomédicas, Universidad de Colima, México. Animals were housed in standard photoperiod conditions (12 h light /12 h dark), a 22 ± 2°C environmental temperature, and fed *ad libitum* with water and rodent food (200 mg iron/kg diet) (Harlan Laboratories, Inc. Stoughton Rd, USA; standard control diet) (Table 1). The experimental design of the present study comprised three components. The first involved experiments using low-iron and control diets, for healthy and diabetic rats. The second step was to conduct experiments in diabetic rats, using a high-iron diet with- or without subcutaneous capsaicin and healthy rats with high iron-diet. Finally, studies were performed using diabetic rats alone fed with a high-iron diet plus oral capsaicin, a standard control diet (Harlan Laboratories, Inc. Stoughton Rd, USA, 200 mg iron/kg diet), or a high-iron diet without capsaicin (Fig 1).

**Table 1:** Standard control diet^¥^. The high-iron diet consists of 200 mg iron/kg diet (standard control diet) plus 3 mg polymalthosed iron, administered orally.

**Macronutrients**	
Crude proteins	18.6%
Fat (ether extract)	6.2%
Carbohydrate (available)	44.2%
Crude fiber	3.5%
Neutral detergent fiber	14.7%
Ash	5.3%
Energy Density	3.1 Kcal /g
Calories from protein	24.0%
Calories from fat	18.0%
Calories from carbohydrate	58.0%
**Minerals**	
Calcium	1.0%
Phosphorus	0.7%
Sodium	0.2%
Potassium	0.6%
Chloride	0.4%
Magnesium	0.2%
Zinc	70 ppm
Manganese	100 ppm
Copper	15 ppm
Iodine	6 ppm
**Iron**	**200 ppm**
Selenium	0.23 ppm

^¥^Harlan Rodent Diet Catalog #2018.

When diabetes was successfully induced in rats (fasting glucose ≥ 200 mg/dL), interventional diets were applied. Low-iron (low-iron diet 3 mg iron/kg diet) and control diets (100 mg iron/kg diet) for healthy and diabetic rats were commenced simultaneously. All experimental protocols and animal management were in accordance with the ethical standards of the Mexican Official Norm technical specifications for the production, care, and use of laboratory animals (NOM-062-ZOO-1999); additional recommendations in the care and use of laboratory animals were from the National Institutes of Health. The condition of the animals was monitored daily. The reporting of this animal research follows the ARRIVE guidelines (S1 File) [19].

### Method of Euthanasia

Rats were euthanized without pain or distress by increasing anesthesia doses (intraperitoneal lethal doses with Pentobarbital Sodium, Pets pharm Mexico). The Ethics Committee from the Universidad de Colima approved all protocols (2011–09).

### The experimental induction of diabetes

To induce diabetes, rats received a single intraperitoneal administration dose of 45 mg/kg body weight of streptozotocin (STZ, Sigma-Aldrich CO. St. Louis MO, USA) [20]. STZ damages pancreatic beta cells, which alters serum glucose. A fasting blood glucose measurement of higher or equal to 200 mg/dL was used to confirm a diabetic state [20,21]. Diseased rats, or those aged less than two months, were excluded from our study.

### Experimental protocol

#### Low-iron diet

The low-iron diet (D03072501) contained 3 mg/kg diet (Research Diets, Inc. New Brunswick, NJ, USA); the interventional control diet (D12050203) contained iron at 100 mg/kg diet (Research Diets, Inc.) (Table 2). These diets were similar to those used by Minamiyama et al. [14]; their low-iron diet contained 3.2 mg/kg diet, and the control diet, 100.9 mg/kg diet. Each diet was used for eight weeks [22].

**Table 2:** Diets used in the low–iron diet experiments. These diets were similar to those used by Minamiyama [14]. See Discussion.

Diet Catalog		D03072501^®^			D12050203^€^	
	g%	kcal%				
Protein	20.3	20.3		20.3	20.3	
Carbohydrate	63.9	63.9		63.9	63.9	
Fat	7	15.8		7	15.8	
Kcal/gm	4			4		
**Ingredient**	**g**	**Kcal**	**ppm Iron**	**g**	**Kcal**	**ppm Iron**
Casein	200	800	1.7	200	800	1.7
L-Cystine	3	12	0	3	12	0
Corn Starch	397.486	1589.944	0.4	397.486	1589.944	0.3974
Maltodextrin 10	132	528	0.11	132	528	0.1056
Sucrose	100	400	0,08	100	400	0,08
Avicel, PH101	50	0	0.015	50	0	0.015
Soybean Oil	70	630	0	70	630	0
t-Butylhydroquinone	0.014	0	0.00014	0.014	0	0.00014
Mineral Mix S18706 (no added iron)	35	0	0.56	35	0	0.56
**Ferric Citrate (17% Iron)**	0	0	0	0.5715	0	97.155
Vitamin Mix V10037	10	40	0.009	10	40	0.009
Choline Bitartrate	2.5	0	0.0005	2.5	0	0.0005
Red Dye, FD&C#40	0.05	0	0.0065	0	0	0
Blue Dye, FD&C#1	0	0	0	0.05	0	0.0033
Total	1000.05	4000	**2.9**	1000.622	4000	**100**

^®^Low–Iron diet 3mg/kg diet,

^€^ Control diet 100mg /kg diet Research Diets.

#### High-iron diet, with or without capsaicin

The standard control diet contained iron at 200 mg/kg diet (Harlan Laboratories, Inc.). Rats were divided into three groups: two (groups 1 and 2) with STZ-induced diabetes, with one healthy group (group 3). All groups were fed with a standard control diet (Harlan Laboratories, Inc.), with iron-regulated diets as described below. Only one group of STZ-induced diabetic rats received capsaicin via a subcutaneous route (1 mg/kg body weight/day); this treatment was of four weeks duration, to enable comparison with previous studies [23,24]. No adverse effects were observed. To explore the role of diabetes on hemoglobin, in the context of a standard control diet and a high-iron diet with or without capsaicin, we used three groups of rats: 1) one group of diabetic rats fed a standard diet (control); 2) a second group of diabetic rats fed with a high-iron diet, and 3), a third group of diabetic rats fed a high-iron diet plus capsaicin, administered by oral gavage at a dose of 1 mg/kg body weight/day for four weeks.

#### Iron administration

Polymaltose iron (Takeda, Mexico, SA de CV, under license of Vifor International, Inc. Switzerland) was administered by oral gavage (Industrial Medical Plastica Silice SA de CV, Mexico) at a dose of 3 mg iron/kg body weight/day for four weeks; a dose proven to cause a significant accumulation of iron [25]. This treatment was used for rats on the high-iron diet (both healthy and diabetic groups).

#### Capsaicin administration

Capsaicin (Sigma-Aldrich, St. Louis, MO, USA) was dissolved in 10% Tween 80 (Sigma-Aldrich, St. Louis, MO, USA) and 10% ethanol. We added a 0.9% saline solution [26] to this mix, which was administered immediately, subcutaneously, at a dose of 1 mg/kg body weight/day [18], over four weeks. For oral use, capsaicin was dissolved in saline solution and immediately administered using an orogastric catheter (Industrial Medical Plastica Silice SA. de CV., Mexico).

### Biochemical measurements

Hemoglobin concentrations were determined in blood samples drawn before and after low- or high-iron diet regimens, using a portable hematoflourometer (Hb201; HemoCue, Ängelholm, Sweden), after 12 h fasting. Similarly, to previous studies [27], we used hemoglobin to assess iron deficiency or iron supplementation (in the high-iron diet). Measurements for glucose were made after 12 h fasting. The oral glucose tolerance test (OGTT) was used in conjunction with the low-iron diet and glucose (2 g/kg body weight), which was administered by oral gavage to the rats, with blood samples drawn at 0, 30, 60, and 120 min. Glucose concentrations were determined with an Accu-Check Active auto-analyzer (Roche, Mannheim, Germany). For the insulin tolerance test (ITT), rats received a subdermal application of insulin (Eli Lilly and Company, Indianapolis, In, USA) (0.1 UI/kg body weight), with blood samples drawn after 0, 5, 15, and 30 min. The data were expressed as area under the curve (AUC) of the OGTT and ITT. The blood glucose concentrations were evaluated using an Accu-Check Active auto-analyzer (Roche, Mannheim, Germany). Blood cholesterol and triglyceride levels were determined in blood samples after 12 h fasting [28–30] with the Accutrend Plus auto-analyzer (Roche, Mannheim, Germany). According to the manufacturer, this instrument had an intra-assay precision of 3.7% for total cholesterol, and 3.4% for triglycerides. Using controls, we calibrated the intra-assay precision as 5% for total cholesterol, and 2.4% for triglycerides.

### Statistical analyses

We used descriptive statistical analyses. The expressed variables were reported as medians with standard error; p-values < 0.05 were considered statistically significant. The Stata software (version 11, StataCorp LP, USA) was used to perform our analyses. We calculated the area under the curve (AUC) for ITT and OGTT tests using the mathematical TAI model [31]. Paired Student’s t-test were used to assess differences in mean values at the beginning and end of each intervention. The Student t-test for independent samples was used to assess differences in the mean values recorded for dietary intervention and controls diets for rats with experimentally induced diabetes. The same analyses were applied to compare mean values at the beginning and end of the intervention, and to compare the mean values between healthy groups of animals. ANOVA was used to assess differences in mean values for high-iron dietary groups.

## Results

### Effects of low dietary iron on diabetic rats

After 8 weeks of either the control diet, or dietary intervention, both groups of rats with STZ-induced diabetes exhibited increased fasting glucose compared with initial values. Rats on the low-iron diet expressed an initial mean fasting glucose concentration of 344.3 ± 15.4 mg/dL, which increased to 495.5 ± 15.3 mg/dL after 8 weeks on the diet (p < 0.001). Rats on the control diet had an initial mean fasting glucose concentration of 322.0 ± 30.2 mg /dL, which increased to 542.8 ± 28.1 mg /dL after 8 weeks (p < 0.001). Fasting glucose levels at the end of the 8-week diet period were lower in the low-iron cohort compared to animals on the control diet (see Table 3), but these differences were not found to be significant (p = 0.17).

**Table 3:** Diabetic and healthy rats fed with low and control iron diet. p value from paired Student t-test at the beginning and end of intervention. Values are mean ± standard error of the mean. Two rats died during the study. Initial refers to values before intervention. Final refers to values after 8 weeks of dietary intervention. The paired Student t-test was calculated with the lower n indicated at the top of the Table.

	Diabetic rats	Diabetic rats	Healthy rats	Healthy rats
	Low-iron	Control diet	Low-iron	Control diet
Variable	3 mg of iron/Kg diet	100 mg of iron/Kg diet	3 mg of iron/Kg diet	100 mg of iron/Kg diet
	n = 8	n = 8	n = 11	n = 11
**Hemoglobin (g/dL)**				
**Initial**	14.9 ± 0.5	15.9 ± 0.2	16.1 ± 0.8	16.0 ± 0.5
**Final**	13.9 ± 0.3	15.6 ± 0.3	12.2 ± 0.2**	16.2 ± 0.3
**Cholesterol (mg/dL)**				
**Initial**	162.6 ± 1.6	166.0 ± 1.4	180.0 ± 7.4	172.1 ± 1.4
**Final**	149.2 ± 0.2*	162.7 ± 3	149.4 ± 1.9*	167.2 ± 4.1
**Triglycerides (mg/dL)**				
**Initial**	123.1 ± 5.8	119.0 ± 3.1	179.9 ± 7.1	185.1 ± 6.3
**Final**	102.8 ± 6.9*	129.8 ± 2.8*	115.0 ± 2.8**	188.1 ± 7.3
**Fasting Glucose (mg/dL)**				
**Initial**	344.3 ± 15.4	322.0 ± 30.2	80.7 ± 3.0	77.5 ± 3.8
**Final**	495.5 ± 15.3**	542.8 ± 28.1**	83.6 ± 2.1	86.9 ± 2.5*
**Area under the curve of OGTT (mg/dL/min)**				
**Initial**	44733±1134.3	30039±3061.7	10662.2±173.9	10472.7±191.4
**Final**	61698.7±4929.3*	64668.7±1721.1**	10786.3±251.6	12432.2 ±305.3**
**Area under the curve of ITT (mg/dL/min)**				
**Initial**	7360.25 ± 673.5	8414 ± 931.7	1883 ± 60.7	1556.8 ± 52.6
**Final**	12523.4 ± 569.4**	14099.6 ±1034.9**	1390 ± 46.03**	1941.36 ± 86.1**
**Weight (g)**				
**Initial**	272.3±9.5	267.5±5.1	261.21±6.5	257±7.4
**Final**	232.8±11.9*	227.2±6.4**	336.8±6.1**	328.9±8.6**

**Fig 2:**
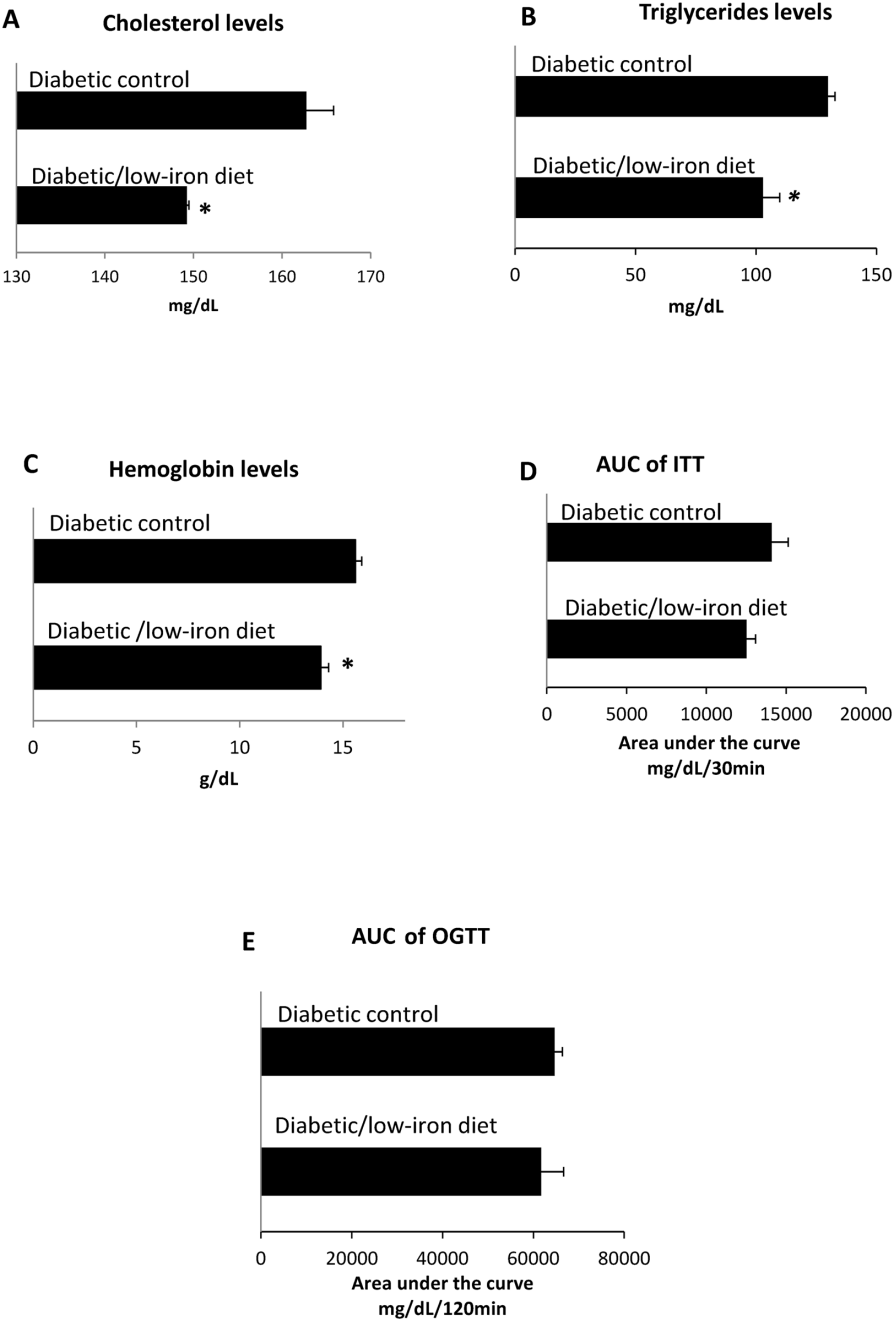
Effects of a low-iron diet on rats with STZ-induced diabetes. Diabetic rats were analyzed after 8 weeks on a low-iron diet (n = 8), or a control diet (n = 8). (A) Cholesterol levels. (B) Triglyceride levels. (C) Hemoglobin levels. (D) Area under insulin curve. (E) Area under glucose tolerance curve. Bars represent the mean and standard error; p-values assessed with Student’s t-test; *p < 0.05.

**Fig 3:**
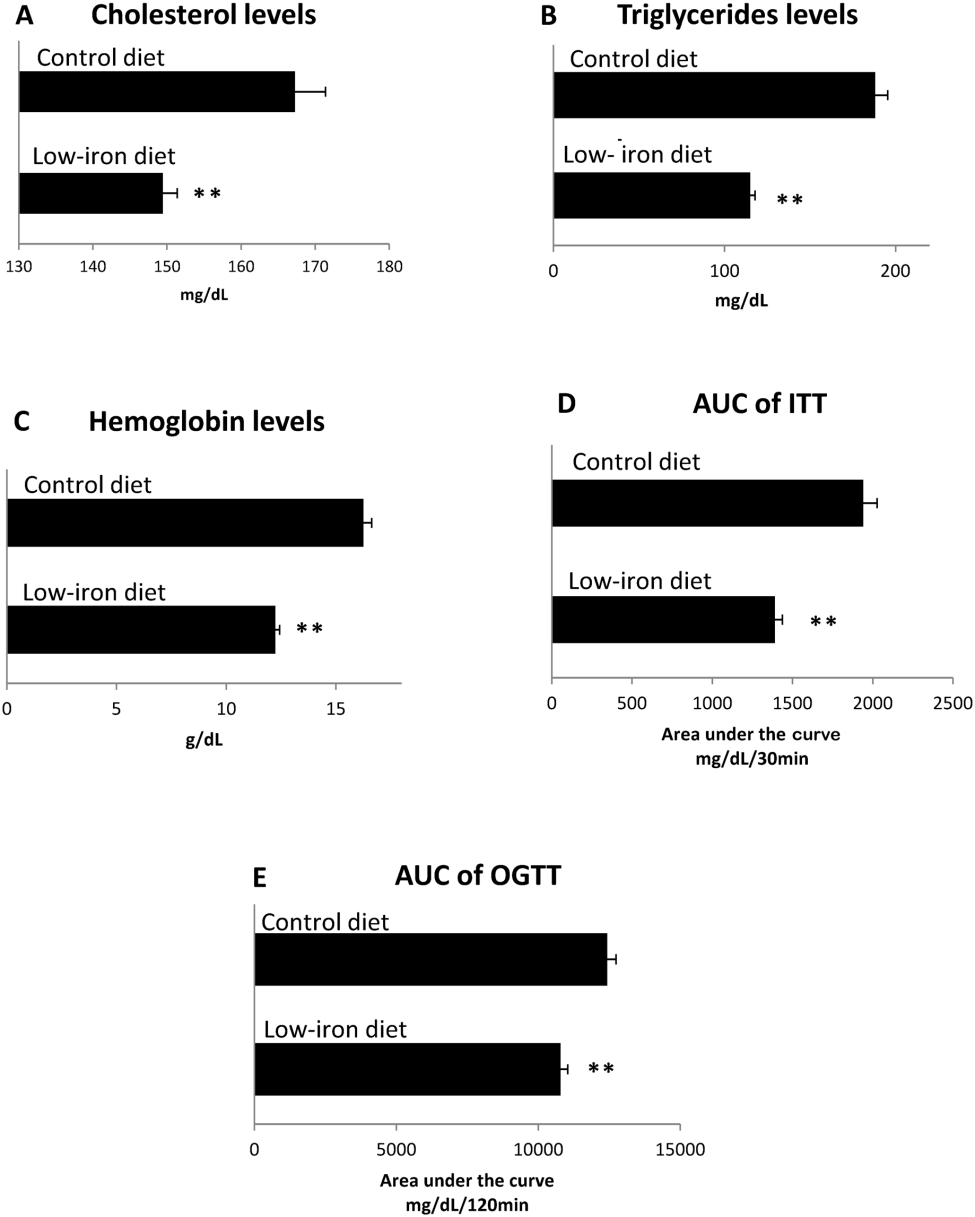
Effects of a low-iron diet on healthy rats. Healthy rats were analyzed after 8 weeks on a low-iron diet (n = 10), or control diet (n = 10). (A) Cholesterol levels. (B) Triglyceride levels. (C) Hemoglobin levels. (D) Area under the insulin tolerance test curve. E) Area under glucose tolerance curve. Bars represent the mean and standard error; p-values assessed with Student’s t-test; *p< 0.05, **p< 0.001.

*p< 0.05,

** p< 0.001, paired Student t-test. Look for cross group comparison in Figs 2 and 3.

The AUC of OGTT in diabetic rats was not statistically different when comparing the low-iron *vs*. control diet groups (p = 0.2) (Table 3, Fig 2E). Similar results were obtained for diabetic rats using the AUC of ITT, when comparing data for the eight-week low-iron *vs*. control diets (Table 3, Fig 2D). Significant reductions in cholesterol and triglycerides were found after 8-weeks of the low-iron diet, compared with rats on a control diet (Fig 2A and 2B). As expected, hemoglobin levels fell significantly in the group that received the low-iron diet, compared with the control group. After 8 weeks, mean hemoglobin levels differed significantly between the low-diet and control diet groups (Table 3; p < 0.05) (Fig 2C). These effects could not be explained by changes in body weight, as this was not substantially altered at the end of the study (232.8 ± 11.9 *vs*. 227.2 ± 6.4 g for diabetic rats on either the low-iron or control diet, respectively (p = 0.6); Table 3).

### Effects of low dietary iron on healthy rats

Cholesterol and triglyceride concentrations were significantly lower (Fig 3A and 3B, Table 3) in the dietary intervention group than in the control diet group, following 8 weeks (p < 0.001) (cholesterol) and p < 0.001 (triglycerides). As expected, after 8 weeks of the low-iron diet, hemoglobin levels in healthy rats decreased from 16.1 ± 0.8 to 12.2 ± 0.2 (p < 0.001). Final hemoglobin concentrations were significantly lower in the dietary intervention group than in the control group (p < 0.001; Fig 3C, Table 3). The areas under the ITT curves were 1390 ± 46.03 mg/dL/min for the intervention group, and 1941 ± 86.1 mg/dL/min for the control group (p < 0.05; Fig 3D, Table 3). At the end of the eight-week diet, the areas under the OGTT curves of the dietary intervention group exhibited a higher glucose tolerance than the control diet group (10786.3 ± 251.6 mg/dL/min *vs*. 12432.2 ± 305.3 mg/dL/min, respectively; p < 0.001; Table 3, Fig 3E).

### Effects of a high-iron diet, with or without capsaicin, on cholesterol, triglyceride, and hemoglobin concentrations

**Fig 4:**
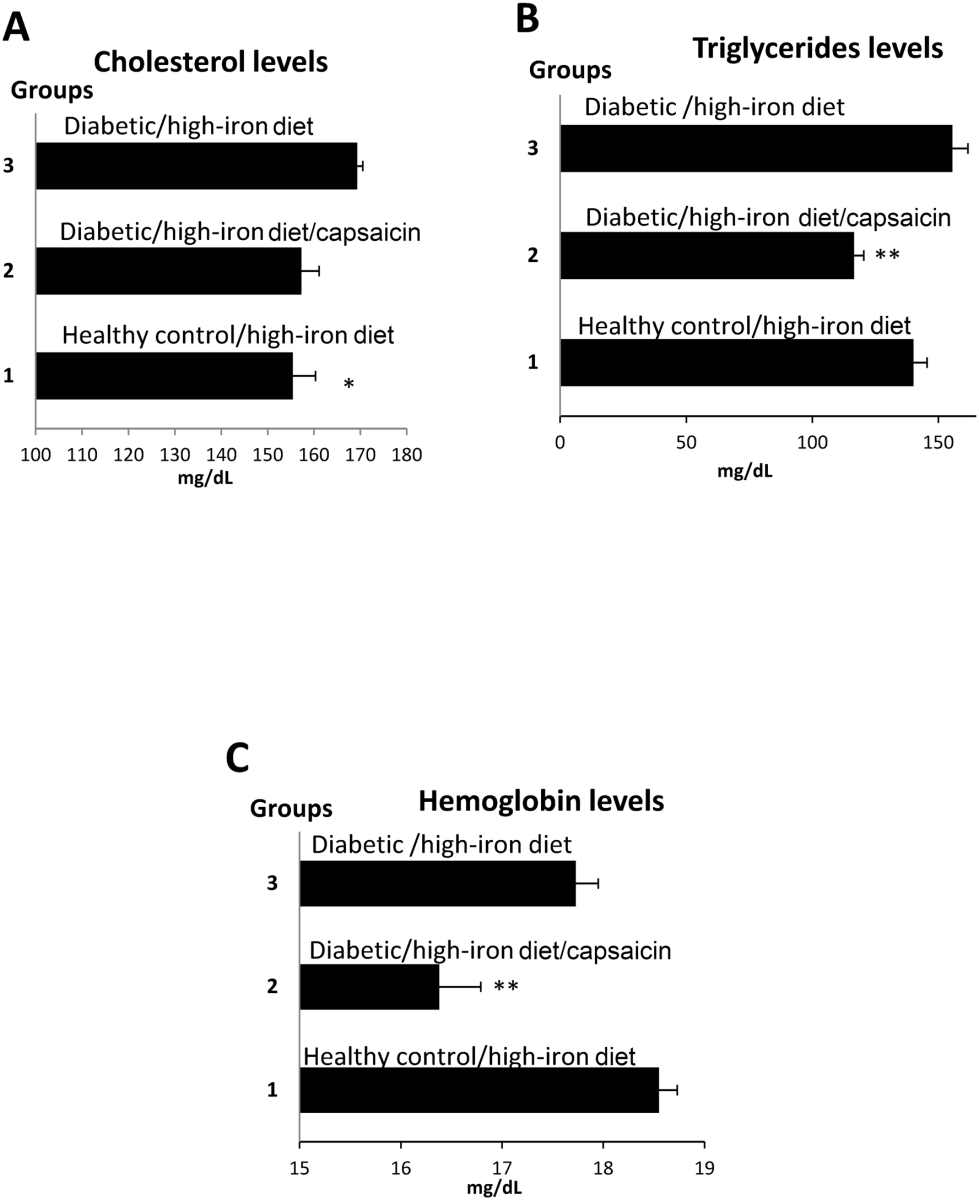
Effects of a high-iron diet, with or without capsaicin, on rats with STZ-induced diabetes. Diabetic and healthy rats were analyzed after 4 weeks on a high-iron diet (n = 10), or a high-iron diet plus capsaicin (3mg/kg body weight/day) (n = 10) (A) Cholesterol levels. (B)Triglyceride levels. (C) Hemoglobin levels. Bars represent the mean and standard error; p- values assessed with ANOVA. For (A) p < 0.05 when comparing group 1 *vs*. groups 2, and 3; for (B) p < 0.001 when comparing group 2 *vs*. group 1, and 3 (C) p < 0.001 when comparing group 2 *vs*. groups 1, and 3. ** p < 0.001, *p < 0.05.

Mice with a form of hereditary iron overload (hemochromatosis) are more likely to develop diabetes [3]. With this in mind, we investigated whether a high iron diet, with or without capsaicin, could alter glucose, cholesterol, triglyceride, or hemoglobin levels. After four weeks of a high-iron diet, cholesterol was significantly increased in diabetic rats versus healthy rats fed the high-iron diet (p < 0.05) (Fig 4A Table 4); triglycerides did not increase significantly (Fig 4B, Table 4). However, cholesterol levels in diabetic rats fed the high-iron diet could be reduced (p < 0.05) by the administration of subcutaneous capsaicin (at a dose of 1 mg/kg body weight/day) to levels comparable with those seen in healthy rats fed the high-iron diet (Fig 4A, Table 4). Capsaicin also significantly reduced triglyceride (p < 0.001) and hemoglobin (p < 0.001) concentration, compared to either the diabetic or healthy test groups fed a high-iron diet (Table 4, Fig 4B and 4C), although glucose levels were not significantly decreased (Table 4).

**Table 4:** Diabetic and healthy rats fed with high iron diet plus capsaicin. p value from paired Student t-test at the beginning and at the end of intervention. The dose of capsaicin used was 1 mg/kg body weight/day administered subcutaneously. Initial refers to values before intervention. Final refers to values after 4 weeks of intervention. To confirm the effects of diabetes on hemoglobin in the high-iron group, an additional group of seven rats were tested; those data have been added ^£^n = 17. The paired Student t-test was calculated with the lower n indicated at the top of the Table. In diabetic rats plus capsaicin the differences between initial and final values corresponds to death of one rat. In healthy rats with high iron the missing values at the beginning were due to technical difficulties in the measurements.

Variable	Diabetic rats High-iron diet plus Capsaicin	Diabetic rats High-iron diet	Healthy rats High-iron diet
	n = 9	n = 10	n = 8
**Hemoglobin (g/dL)**			
**Initial**	19.2±0.3	18.3±0.4^£^	18.3±0.4
**Final**	16.3±0.4**	17.7±0.2^£^	18.55±0.1
**Cholesterol (mg/dL)**			
**Initial**	152.5±1.7	154.4±2.8	150.2±0.9
**Final**	157.3±4.2	169.4±1.2**	154±5.1
**Triglycerides(mg/dL)**			
**Initial**	103.3±9.1	109±12.5	135.8±19.7
**Final**	116.5±3.8	155.6±6.1*	140.1±5.3
**Fasting Glucose (mg/dL)**			
**Initial**	409.3±30.5	397.5±30.6	90.6±2.6
**Final**	393.6±43.7	412.5±17.3	92.9±2.1

*p<0.05,

** p<0.001, paired Student t-test.

Look for cross group comparison in Fig 4.

Previous studies in patients with diabetes showed that renal dysfunction modifies hemoglobin levels (5,6). We have studied the dual effects of diabetes and dietary iron on hemoglobin concentration in experiments conducted with diabetic rats fed a standard control diet (Harlan). In terms of hemoglobin concentration, tend to be lower, but not statistically significant differences were found for values acquired initially, versus those after four weeks of treatment (17.6 ± 0.5 vs. 17.3 ± 0.5 g/dL respectively; p = 0.8; n = 7). This outcome was also seen for diabetic rats fed with a high-iron diet (18.3 ± 0.4 vs. 17.7 ± 0.2 g/dL for initial and 4 weeks of hemoglobin levels, respectively, p = 0.08; Table 4). However, for diabetic rats fed with a high iron diet plus oral capsaicin, hemoglobin levels were significantly reduced when comparing the initial values with those after four weeks of treatment (17.4 ± 0.4 vs. 15.8 ± 0.4 g/dL, respectively; p = 0.02; n = 7). These results show that oral capsaicin significantly reduced the levels of hemoglobin, replicating the effects seen with subcutaneous administration (Table 4).

## Discussion

The results of this study using a rat chow diet showed that a low-iron diet could reduce total cholesterol and triglyceride levels to the same degree in both healthy and diabetic rats. In addition, the low-iron diet was associated with a significantly improved insulin and glucose tolerance in healthy rats.

One mechanistic insight for the role of dietary iron has been previously published by Mehdad et al. 2015; who showed that reduced dietary iron enhanced insulin receptor expression in skeletal muscle [32]. On the other hand, our results agree with those of Minamiyama et al. [14], in which the effects of iron depletion on lipid profiles and glycosylated hemoglobin (HbA1c) levels were examined. Otsuka Long-Evans Tokushima Fatty (OLETF) rats were used in that study [12], age-paired with Long-Evans Tokushima Otsuka (LETO) rats. Low dietary iron (3.2 mg iron/kg diet), or phlebotomy, were used to reduce iron levels in the blood, with comparisons made with rats fed a control iron diet (100.9 mg iron/kg diet). The results of the present study also agree with an investigation using obese mice (ob/ob, *lep-/-*), conducted by Cooksey et al. [12]. At the end of that study, mice that received a low-iron diet exhibited a diminution in triglyceride and cholesterol levels, and increased insulin sensitivity, relative to controls. One possible mechanism for these effects was suggested by a study by Kamei et al. [33], in which the transcriptional response to the experimental induction of anemia was studied in Sprague Dawley rats. In that study, changes in the transcription of genes involved in metabolism were identified. With respect to our data collected with low dietary iron, we could speculate that altered gene expression, possibly acting directly on cholesterol and triglyceride synthesis, could be relevant. However, we should emphasize that in our study, the low-iron diet was moderate, such that anemia was avoided (< 10 g hemoglobin/dL). Additional studies would be needed to establish the timeframe over which a low-iron diet would be needed to induce anemia. On the other hand, at the end of eight-weeks, for the diabetic rats there exists a significant loss in weight. Previous studies in type 2 diabetic patients showed weight loss when these patients failed to receive treatment for diabetes control [34]. More experiments are now needed to determine if the model we use corresponds more accurately to type 2 or type 1 of diabetes.

The experimental designs of previous studies have differed from ours mainly in terms of the animal models used. For example, both ob/ob mice and OLETF rats are natural models of obesity and develop hyperglycemia later in life; both models are genetically predisposed to obesity. A possible explanation for the discrepancies between our data and previous studies is that they were performed using animal models of type 2 diabetes with hyperinsulinemia, while the present study was performed in an animal model of hypoinsulinemia. Our findings of a lack of any dietary effect on either fasting glucose concentration may be related to exposure time. In contrast, rats fed with a high-iron diet exhibited increased in total cholesterol level. To provide mechanistic context, some authors have linked chronic dietary iron overload and lipid peroxidation, with impaired plasma lipid transport, and therefore increased cholesterol [9]. Another credible possibility is that excess stored iron might cause oxidative stress, with reactive oxygen species subsequently damaging cell membranes, lipids, and proteins. The resultant lesions could damage tissue, and organs, including the liver and pancreas [35,36]. Graham et al. [37] also suggested that hepatic iron overload could increase hepatic cholesterol biosynthesis. Clearly, the mechanistic interactions between iron and cholesterol remain unclear.

To summarize, we now show that a low-iron diet reduced triglyceride and cholesterol levels in healthy rats, and in rats with experimentally induced diabetes. The low-iron diet also improved insulin and glucose tolerance in healthy rats. In contrast, these same biochemical parameters were worsened in the context of anemia (<10 g hemoglobin/dL) [38], which is clinically relevant given that both anemia, and hemochromatosis, have been shown to increase morbidity and mortality through heart failure and thrombosis [39,40].

Our results suggest that a low-iron diet reduce the dyslipidemia, and improve insulin and glucose tolerance in healthy rats. Showing that context is important, Ikeda et al. [22] have also demonstrated that a low-iron diet prevented diabetic nephropathy in diabetic rats, which was partly attributable to reduced oxidative stress. With respect to capsaicin, Prakash and Srinivasan [17] reported that acute administration (60 min) of capsaicin intake enhanced the intestinal uptake of iron in segments of isolated intestine. While previous studies failed to find any substantial effects on cholesterol metabolism that could be attributed to acute capsaicin intake (in conjunction with a standard diet), [41] the oxidation of low-density lipoprotein (in rats) has been reported [42]. We now demonstrate that capsaicin reduces hemoglobin when administered as part of a high-iron diet [5]. As previously mentioned, diabetes per se may modify hemoglobin concentration, mainly via renal dysfunction which alters erythropoietin synthesis, and subsequently hemoglobin level [5, 6]. Our data show that the reduction in hemoglobin level achieved using both oral and subcutaneous capsaicin, were greater than that achieved by the diabetic state alone. Additionally, the levels of hemoglobin in diabetic rats, on both the high-iron and standard control diets (Harlan), were not significantly different. Further research is now required to investigate the mechanisms for capsaicin’s action on hemoglobin, lipid metabolism, and iron regulation, as well as possible therapeutic uses.

## Conclusions

Our results suggest that a low-iron diet could improve lipid homeostasis in diabetes, and that capsaicin reduces hemoglobin levels in rats fed a high-iron diet.

## Supporting Information

S1 File NC3Rs ARRIVE Guidelines Checklist-FILLED FINAL.pdf. (PDF)
